# Inflammatory responses and inflammation-associated diseases in organs

**DOI:** 10.18632/oncotarget.23208

**Published:** 2017-12-14

**Authors:** Linlin Chen, Huidan Deng, Hengmin Cui, Jing Fang, Zhicai Zuo, Junliang Deng, Yinglun Li, Xun Wang, Ling Zhao

**Affiliations:** ^1^ College of Veterinary Medicine, Sichuan Agricultural University, Wenjiang, Chengdu 611130, China; ^2^ Key Laboratory of Animal Diseases and Environmental Hazards of Sichuan Province, Sichuan Agriculture University, Wenjiang, Chengdu 611130, China

**Keywords:** inflammation, inflammatory signaling pathways, chemokines, cytokines, organ diseases

## Abstract

Inflammation is a biological response of the immune system that can be triggered by a variety of factors, including pathogens, damaged cells and toxic compounds. These factors may induce acute and/or chronic inflammatory responses in the heart, pancreas, liver, kidney, lung, brain, intestinal tract and reproductive system, potentially leading to tissue damage or disease. Both infectious and non-infectious agents and cell damage activate inflammatory cells and trigger inflammatory signaling pathways, most commonly the NF-κB, MAPK, and JAK-STAT pathways. Here, we review inflammatory responses within organs, focusing on the etiology of inflammation, inflammatory response mechanisms, resolution of inflammation, and organ-specific inflammatory responses.

## INTRODUCTION

Inflammation is the immune system's response to harmful stimuli, such as pathogens, damaged cells, toxic compounds, or irradiation [1], and acts by removing injurious stimuli and initiating the healing process [2]. Inflammation is therefore a defense mechanism that is vital to health [3]. Usually, during acute inflammatory responses, cellular and molecular events and interactions efficiently minimize impending injury or infection. This mitigation process contributes to restoration of tissue homeostasis and resolution of the acute inflammation. However, uncontrolled acute inflammation may become chronic, contributing to a variety of chronic inflammatory diseases [4].

At the tissue level, inflammation is characterized by redness, swelling, heat, pain, and loss of tissue function, which result from local immune, vascular and inflammatory cell responses to infection or injury [5]. Important microcirculatory events that occur during the inflammatory process include vascular permeability changes, leukocyte recruitment and accumulation, and inflammatory mediator release [2, 6].

Various pathogenic factors, such as infection, tissue injury, or cardiac infarction, can induce inflammation by causing tissue damage. The etiologies of inflammation can be infectious or non-infectious (Table 1). In response to tissue injury, the body initiates a chemical signaling cascade that stimulates responses aimed at healing affected tissues. These signals activate leukocyte chemotaxis from the general circulation to sites of damage. These activated leukocytes produce cytokines that induce inflammatory responses [7].

**Table 1:** Etiology of inflammation

Non-infectious factors	Infectious factors
Physical: burn, frostbite, physical injury, foreign bodies, trauma, lionizing radiation Chemical: glucose, fatty acids, toxins, alcohol, chemical irritants (including fluoride, nickel and other trace elements) Biological: damaged cells Psychological: excitement	Bacteria viruses other microorganisms

## INFLAMMATORY RESPONSE MECHANISMS

The inflammatory response is the coordinate activation of signaling pathways that regulate inflammatory mediator levels in resident tissue cells and inflammatory cells recruited from the blood [8]. Inflammation is a common pathogenesis of many chronic diseases, including cardiovascular and bowel diseases, diabetes, arthritis, and cancer [9]. Although inflammatory response processes depend on the precise nature of the initial stimulus and its location in the body, they all share a common mechanism, which can be summarized as follows: 1) cell surface pattern receptors recognize detrimental stimuli; 2) inflammatory pathways are activated; 3) inflammatory markers are released; and 4) inflammatory cells are recruited.

### Pattern recognition receptor activation

Microbial structures known as pathogen-associated molecular patterns (PAMPs) can trigger the inflammatory response through activation of germline-encoded pattern-recognition receptors (PRRs) expressed in both immune and nonimmune cells [10, 11]. Some PRRs also recognize various endogenous signals activated during tissue or cell damage and are known as danger-associated molecular patterns (DAMPS) [11]. DAMPs are host biomolecules that can initiate and perpetuate a non-infectious inflammatory response [12]. Disrupted cells can also recruit innate inflammatory cells in the absence of pathogens by releasing DAMPs [13].

**Figure 1:**
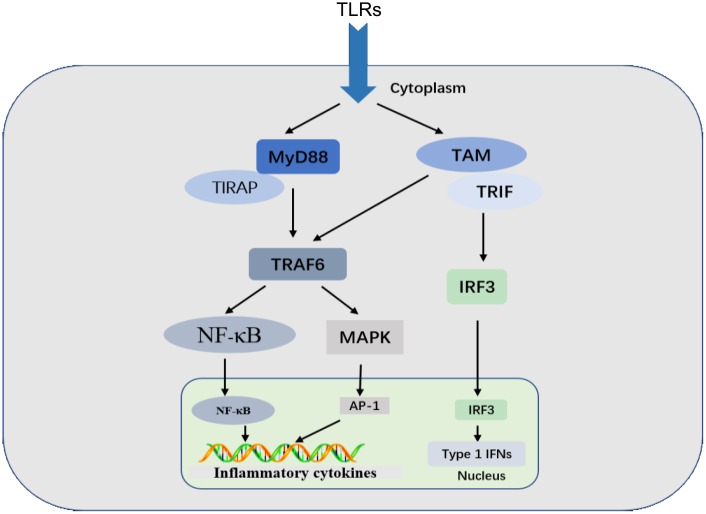
TLR signaling MyD88-dependent and TRIF-dependent pathways are shown. Signaling through TLRs activates intracellular signaling cascades that lead to nuclear translocation of AP-1 and NF-κB or IRF3, which regulates the inflammatory response.

Classes of PRR families include the Toll-like receptors (TLRs), C-type lectin receptors (CLRs), retinoic acid-inducible gene (RIG)-I-like receptors (RLRs), and NOD-like receptors (NLRs) [5]. TLRs are a family of highly conserved, mammalian PRRs that participate in the activation of the inflammatory response [14]. More than ten members of the TLR family have been identified, and TLRs are the most well-studied of the known PRRs [15]. Transmission of PAMPs and DAMPs is mediated by myeloid differentiation factor-88 (MyD88) along with TLRs. Signaling through TLRs activates an intracellular signaling cascade [16, 17] that leads to nuclear translocation of transcription factors, such as activator protein-1 (AP-1) and NF-κB or interferon regulatory factor 3 (IRF3) (Figure 1). DAMPs and PAMPs share receptors, such as TLR4, suggesting similarities between infectious and noninfectious inflammatory responses [18, 19].

### Activation of inflammatory pathways

Inflammatory pathways impact the pathogenesis of a number of chronic diseases, and involve common inflammatory mediators and regulatory pathways. Inflammatory stimuli activate intracellular signaling pathways that then activate production of inflammatory mediators. Primary inflammatory stimuli, including microbial products and cytokines such as interleukin-1β (IL-1β), interleukin-6 (IL-6), and tumor necrosis factor-α (TNF-α), mediate inflammation through interaction with the TLRs, IL-1 receptor (IL-1R), IL-6 receptor (IL-6R), and the TNF receptor (TNFR) [20]. Receptor activation triggers important intracellular signaling pathways, including the mitogen-activated protein kinase (MAPK), nuclear factor kappa-B (NF-κB), and Janus kinase (JAK)-signal transducer and activator of transcription (STAT) pathways [21–23].

### NF-κB pathway

**Figure 2:**
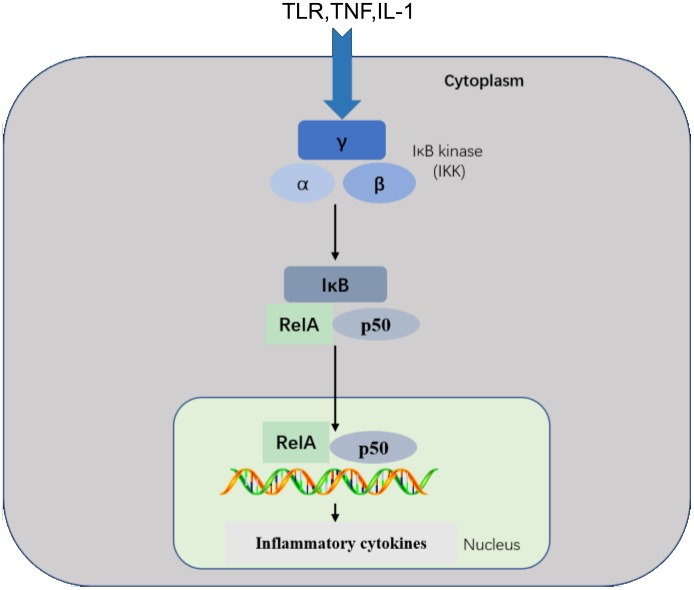
NF-κB pathway This pathway is triggered by TLRs and inflammatory cytokines, such as TNF and IL-1, leading to activation of RelA/p50 complexes that regulate expression of inflammatory cytokines. NF-κB signaling requires IKK subunits. which regulate pathway activation through IκB phosphorylation.

The NF-κB transcription factor plays important roles in inflammatory, immune response, survival, and apoptosis processes [24]. The NF-κB family includes five related transcription factors: P50, p52, RelA (p65), RelB, and c-Rel [25, 26]. NF-κB activity is induced by a range of stimuli, including pathogen-derived substances, intercellular inflammatory cytokines, and many enzymes [27, 28]. Under physiological conditions, IκB proteins present in the cytoplasm inhibit NF-κB [29]. PRRs use similar signal transduction mechanisms to activate IκB kinase (IKK), which is composed of two kinase subunits, IKKα and IKKβ, and a regulatory subunit, such as IKKγ. IKK regulates NF-κB pathway activation through IκB phosphorylation [8]. IκB phosphorylation results in its degradation by the proteasome and the subsequent release of NF-κB for nuclear translocation and gene transcription activation [30]. This pathway regulates pro-inflammatory cytokine production and inflammatory cell recruitment, which contribute to the inflammatory response (Figure 2).

### MAPK pathway

**Figure 3:**
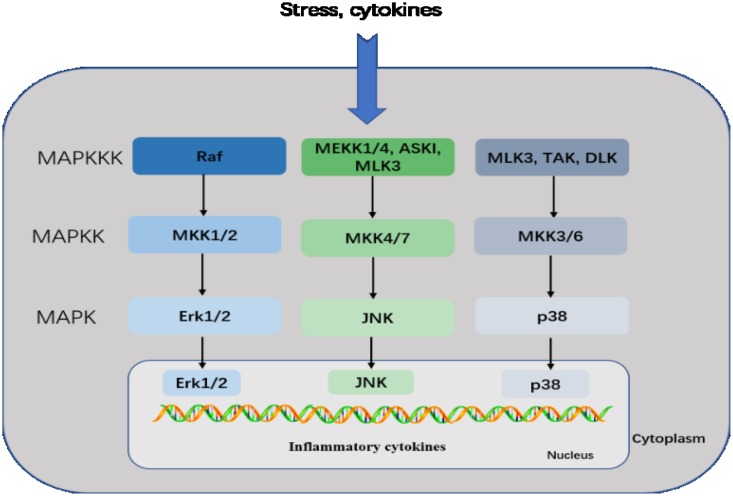
MAPK pathway This pathway mediates intracellular signaling initiated by extracellular stimuli, such as stress and cytokines. MAPKKKs phosphorylate and activate MAPKKs, which in turn phosphorylate and activate MAPKs. The mammalian MAPK family includes Erk1/2, JNK, and p38. In the Erk1/2 pathway, Erk1/2 is activated by MKK1/2, which is activated by Raf. In the JNK pathway, JNK is activated by MKK4/7, which is activated by MEKK1/4, ASK1, and MLK3. In the p38 pathway, p38 is activated by MKK3/6, which is activated by MLK3, TAK, and DLK. Activated MAPKs phosphorylate various proteins, including transcription factors, resulting in regulation of inflammatory responses.

MAPKs are a family of serine/threonine protein kinases that direct cellular responses to a variety of stimuli, including osmotic stress, mitogens, heat shock, and inflammatory cytokines (such as IL-1, TNF-α, and IL-6), which regulate cell proliferation, differentiation, cell survival and apoptosis [31, 32]. The mammalian MAPKs include extracellular-signal-regulated kinase ERK1/2, p38 MAP Kinase, and c-Jun N-terminal kinases (JNK) [33]. Each MAPK signaling pathway comprises at least three components: a MAPK, a MAPK kinase (MAPKK), and a MAPK kinase kinase (MAPKKK). MAPKKKs phosphorylate and activate MAPKKs, which in turn phosphorylate and activate MAPKs [33, 34]. ERKs are generally activated by mitogens and differentiation signals, while inflammatory stimuli and stress activate JNK and p38 [35]. MKK1 and MKK2 activate ERK1/2, MKK4 and MKK7 activate JNK, and MKK3 and MKK6 activate p38. Activation of the MAPKs, including Erk1/2, JNK, leads to phosphorylation and activation of p38 transcription factorsv present in the cytoplasm or nucleus, which initiates the inflammatory response [32, 36] (Figure 3).

### JAK-STAT pathway

**Figure 4:**
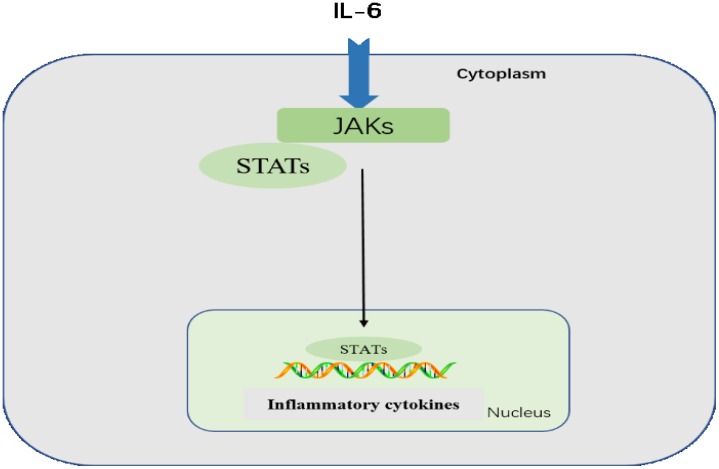
JAK-STAT pathway Following IL-6 binding, signal is transduced by a receptor to activate the JAKs, which then activate STATs. STATs are dephosphorylated in the nucleus, leading to activation of downstream cytokines.

The highly conserved JAK-STAT pathway involves diverse cytokines, growth factors, interferons, and related molecules, such as leptin and growth hormone, and is a signaling mechanism through which extracellular factors can control gene expression [37]. Receptor-associated JAKs are activated by ligands and phosphorylate one other, creating docking sites for STATs, which are latent, cytoplasmic transcription factors. Cytoplasmic STATs recruited to these sites undergo phosphorylation and subsequent dimerization before translocation to the nucleus [38]. Tyrosine phosphorylation is essential for STAT dimerization and DNA binding [39]. Therefore, JAK/STAT signaling allows for the direct translation of an extracellular signal into a transcriptional response. For example, binding of IL-6 family members to plasma membrane receptors activates the JAK-STAT proteins. STAT proteins translocated into the nucleus bind target gene promoter regions to regulate transcription of inflammatory genes (Figure 4) [40].

Dysregulation of NF-κB, MAPK, or JAK-STAT activity is associated with inflammatory, autoimmune, and metabolic diseases, and cancer [41]. Signaling through transcription factors results in secretion of cytokines [42, 43]. Multiple transcription factors regulate a variety of inflammatory genes, such as IL-1, TNF-α, IL-6 [44], colony stimulating factor (CSF), interferons, transforming growth factor (TGF), and chemokines.

### Inflammatory markers

Markers are used in clinical applications to indicate normal versus pathogenic biological processes, and assess responses to therapeutic interventions. Inflammatory markers may be predictive of inflammatory diseases [45–50], and correlate with the causes and consequences of various inflammatory diseases, such as cardiovascular diseases, endothelial dysfunctions, and infection [51, 52]. Stimuli activate inflammatory cells, such as macrophages and adipocytes, and induce production of inflammatory cytokines, such as IL-1β, IL-6, TNF-α, and inflammatory proteins and enzymes. These molecules can potentially serve as biomarkers for diseases diagnosis, prognosis, and therapeutic decision making [53–57].

### Inflammatory cytokines

Cytokines (Table 2) are predominantly released from immune cells, including monocytes, macrophages, and lymphocytes. Pro- and anti-inflammatory cytokines facilitate and inhibit inflammation, respectively. Inflammatory cytokines are classified as ILs, colony stimulating factors (CSF), IFNs, TNFs, TGFs, and chemokines, and are produced by cells primarily to recruit leukocytes to the site of infection or injury [58]. Cytokines modulate the immune response to infection or inflammation and regulate inflammation itself via a complex network of interactions. However, excessive inflammatory cytokine production can lead to tissue damage, hemodynamic changes, organ failure, and ultimately death [59, 60]. A better understanding of how to regulate cytokine pathways would allow for more accurate identification of agent-mediated inflammation and the treatment of inflammatory diseases [58].

**Table 2:** Summary of cytokines and their functions

Cytokine	Family	Main sources	Function
IL-1β	IL-1	Macrophages, monocytes	Pro-inflammation, proliferation, apoptosis, differentiation
IL-4	IL-4	Th-cells	Anti-inflammation, T-cell and B-cell proliferation, B-cell differentiation
IL-6	IL-6	Macrophages, T-cells, adipocyte	Pro-inflammation, differentiation, cytokine production
IL-8	CXC	Macrophages, epithelial cells, endothelial cells	Pro-inflammation, chemotaxis, angiogenesis
IL-10	IL-10	Monocytes, T-cells, B-cells	Anti-inflammation, inhibition of the pro-inflammatory cytokines
IL-12	IL-12	Dendritic cells, macrophages, neutrophils	Pro-inflammation, cell differentiation, activates NK cell
IL-11	IL-6	Fibroblasts, neurons, epithelial cells	Anti-inflammation, differentiation, induces acute phase protein
TNF-α	TNF	Macrophages, NK cells, CD4^+^lymphocytes, adipocyte	Pro-inflammation, cytokine production, cell proliferation, apoptosis, anti-infection
IFN-γ	INF	T-cells, NK cells, NKT cells	Pro-inflammation, innate, adaptive immunity anti-viral
GM-CSF	IL-4	T-cells, macrophages, fibroblasts	Pro-inflammation, macrophage activation, increase neutrophil and monocyte function
TGF-β	TGF	Macrophages, T cells	Anti-inflammation, inhibition of pro-inflammatory cytokine production

### Inflammatory proteins and enzymes

Inflammatory proteins in the blood, including C-reactive protein (CRP), haptoglobin, serum amyloid A, fibrinogen, and alpha 1-acid glycoprotein [61], help restore homeostasis and reduce microbial growth independently of antibodies during trauma, stress, or infection [62]. Abnormal activation of certain enzymes, including high-mobility group box 1 (HMGB1), superoxide dismutase (SOD), glutathione peroxidase (GPx), NADPH oxidase (NOX), inducible nitric oxide synthase (iNOS) and cyclooxygenase (COX)-2, play key roles in the development of inflammation-related diseases, such as cardiovascular disease and cancer [63–66]. For example, extracellular HMGB1 effects may be mediated by activation of TLR-coupled signaling pathways [67]. The primary target of extracellular HMGB1 is TLR4 [68], which triggers MyD88-dependent intracellular signaling cascades involved in activation of the NF-κB and MAPK pathways. This leads to release of such inflammatory cytokines as TNF-α and IL-1β [67]. Inflammatory proteins and enzymes have been used as inflammation, infection, and trauma biomarkers in medicine.

### Other inflammatory markers

Antioxidant defense systems, including antioxidant enzymes, influence oxidative stress. Elevated oxidative stress can induce production of reactive oxygen species (ROS), malondialdehyde (MDA), 8-Hydroxy-2-deoxyguanosine (8-OHdG) and isoprostanes [64, 69], each of which can activate various transcription factors, including NF-κB, AP-1, p53, and STAT. Thus, this cascade can increase expression of genes encoding growth factors, inflammatory cytokines, and chemokines [70]. Oxidative stress is associated with the pathogenesis of multiple diseases, such as cardiovascular disease, cancer, diabetes, hypertension, aging, and atherosclerosis. Therefore, oxidative stress products can also be used as markers of the inflammatory response.

### Cell types in inflammatory responses

The inflammatory response involves a highly coordinated network of many cell types. Activated macrophages, monocytes, and other cells mediate local responses to tissue damage and infection. At sites of tissue injury, damaged epithelial and endothelial cells release factors that trigger the inflammatory cascade, along with chemokines and growth factors, which attract neutrophils and monocytes. The first cells attracted to a site of injury are neutrophils, followed by monocytes, lymphocytes (natural killer cells [NK cells], T cells, and B cells), and mast cells [71–73]. Monocytes can differentiate into macrophages and dendritic cells and are recruited via chemotaxis into damaged tissues. Inflammation-mediated immune cell alterations are associated with many diseases, including asthma, cancer, chronic inflammatory diseases, atherosclerosis, diabetes, and autoimmune and degenerative diseases.

Neutrophils, which target microorganisms in the body, can also damage host cells and tissues [74]. Neutrophils are key mediators of the inflammatory response, and program antigen presenting cells to activate T cells and release localized factors to attract monocytes and dendritic cells [7]. Macrophages are important components of the mononuclear phagocyte system, and are critical in inflammation initiation, maintenance, and resolution [75]. During inflammation, macrophages present antigens, undergo phagocytosis, and modulate the immune response by producing cytokines and growth factors. Mast cells, which reside in connective tissue matrices and on epithelial surfaces, are effector cells that initiate inflammatory responses. Activated mast cell release a variety of inflammatory mediators, including cytokines, chemokines, histamine, proteases, prostaglandins, leukotrienes, and serglycin proteoglycans [76].

Multiple groups have demonstrated that platelets impact inflammatory processes, from atherosclerosis to infection. Platelet interactions with inflammatory cells may mediate pro-inflammatory outcomes. The acute phase response (APR) is the earliest response to infection or injury, and some studies have indicated that platelets induce the APR [77]. After being recruited by inflammatory stimuli, immune cells amplify and sustain the APR by releasing local inflammatory mediators at the site of recruitment.

## RESOLUTION OF INFLAMMATION

To prevent progression from acute inflammation to persistent, chronic inflammation, the inflammatory response must be suppressed to prevent additional tissue damage. Inflammation resolution is a well-managed process involving the spatially- and temporally-controlled production of mediators, during which chemokine gradients are diluted over time. Circulating white blood cells eventually no longer sense these gradients and are not recruited to sites of injury. Dysregulation of this process can lead to uncontrolled chronic inflammation [78]. Inflammation resolution processes that rectify tissue homeostasis include reduction or cessation of tissue infiltration by neutrophils and apoptosis of spent neutrophils, counter-regulation of chemokines and cytokines, macrophage transformation from classically to alternatively activated cells, and initiation of healing [79, 80].

Chronic inflammation occurs when acute inflammatory mechanisms fail to eliminate tissue injury [81], and may lead to a host of diseases, such as cardiovascular diseases, atherosclerosis, type 2 diabetes, rheumatoid arthritis, and cancers [82]. Understanding the common mechanisms that orchestrate dysfunction in the various organ systems will allow for development and production of improved targeted therapies.

## ORGAN-SPECIFIC INFLAMMATORY RESPONSES

Inflammation has long been recognized as a major cause of disease. It is estimated that some 15% of human cancers are associated with chronic infection and inflammation [83]. Acute and chronic inflammation-mediated tissue injury is observed in many organ systems, including the heart, pancreas, liver, kidney, lung, brain, intestinal tract, and reproductive system.

### Heart

Cardiovascular disease, and its underlying pathology, atherosclerosis, is the major cause of death and disability worldwide [84, 85]. By 2030, almost 23.6 million people are projected to die annually from cardiovascular disorders [86, 87]. Inflammatory mediators play key roles in atherosclerosis, from initial leukocyte recruitment through rupture of the atherosclerotic plaque [88–91].Inflammation is also an early event in cardiac stress. Elevated levels of endothelial adhesion molecules and increased inflammatory cytokine and chemokine production and release are observed in affected cardiac tissues [92].

The innate immune system is the primary cardiac defense against pathogens and tissue damage [93]. Myocardial infarction, which commonly results from coronary atherosclerosis and involves acute loss of many myocardial cells, is the most common cause of cardiac injury [94]. Necrotic cardiac cells initiate an inflammatory cascade to clear dead cells and debris from the infarct [95, 96]. Cell death releases intracellular components that activate innate immune mechanisms to initiate an inflammatory response. Endogenous ligands released following injury are recognized as danger signals by cell surface receptors, and activate inflammation [97, 98]. TLR-mediated pathways trigger post-infarction inflammatory responses by activating NF-κB signaling [98–103]. Chemokines recruit inflammatory leukocytes to the infarct, and cytokines promote leukocyte-endothelial cell adhesions [104, 105]. Moreover, TGF-β and IL-10 promote cardiac repair by suppressing inflammation, enhancing myofibroblast phenotypic modulation, and promoting extracellular matrix deposition [106, 107].

Cardiovascular disease is the main cause of death and disability in patients with diabetes mellitus, especially those with type 2 diabetes (T2D), in whom cardiovascular disease occurs 14.6 years earlier on average [108]. About two-thirds of deaths in people with diabetes are due to cardiovascular disease; among these, approximately 40% die from ischemic heart disease, 15% from other forms of heart disease, principally congestive heart failure, and about 10% from stroke [109]. Recent global estimates indicate that over 422 million adults currently live with diabetes, of which over 90% have T2D.

Diabetes is a group of metabolic disorders characterized by sustained high blood sugar levels, and is a major global health challenge, both to individuals and their families, and to healthcare systems [110]. Diabetes complications include heart attack, stroke, kidney failure, limb amputation, blindness, and nerve damage. Diabetes results from either impaired insulin production in the pancreas or body cells not responding to produced insulin [111]. Insulin resistance is defined as decreased insulin-stimulated glucose uptake, and is associated with inactivity, obesity, and aging. Pancreatic islet cells respond to insulin resistance by enhancing insulin secretion and cell mass. However, when islet β-cells are unable to compensate for insulin resistance, insulin deficiency develops, followed by T2D [112], which is increasingly being characterized as an inflammatory disease [113, 114]. Elevated circulating levels of acute-phase proteins, including CRP, fibrinogen, serum amyloid A, plasminogen activator inhibitor, and haptoglobin, along with sialic acid, cytokines, and chemokines, have been observed in patients with T2D. Elevated IL-1β, IL-6, TNF-α, and CRP levels are also predictive of T2D. IL-1 receptor antagonist (IL-1RA) is elevated in obesity and prediabetes prior to T2D onset. Excessive nutrient levels, including those of glucose and free fatty acids, promote insulin resistance. T2D also activates the NF-κB, MAPK, and JAK-STAT pathways, which can each promote tissue inflammation [110, 114, 115]. Metabolic stressors also negatively impact pancreatic islet cells and insulin-sensitive tissues, including adipose tissue, promoting local cytokine and chemokine production and release. At the same time, immune cells, such as mast cells and macrophages, are recruited and contribute to tissue inflammation. Similarly, cytokine and chemokine release from adipose tissues into the circulation promotes further inflammation in other tissues [116].

### Pancreas

Pancreatitis, caused by pancreatic duct obstruction, trypsinogen gene mutation, or alcoholism, is an inflammatory disease of the pancreas [117]. Acute pancreatitis (AP) incidence ranges from 4–45 per 100,000 patients per year and increases annually by approximately 1.3–4.0% in most developed countries. AP is one of the most common gastrointestinal causes for hospitalization in the US, and chronic pancreatitis (CP) is less common than AP. However, CP patients experience chronic abdominal pain and exocrine and/or endocrine insufficiency, leading to reduced quality of life [118]. Pancreatitis is characterized by acinar cell destruction and activation of inflammatory cells, including macrophages, neutrophils, and granulocytes, which secrete inflammatory cytokines [117, 119]. These cytokines further activate pancreatic stellate cells (PSCs) to promote CP [120]. Pancreatitis development requires various molecular pathways, such as NF-κB, MAPK, and JAK-STAT, which play critical roles in inflammatory cell activation during pancreatitis [117].

Pancreatic cancer (PC) remains one of the most lethal of malignancies and a major health burden [121], and is the fourth most common cause of death from cancer in the US [118]. There is a strong link between antecedent CP and PC [122]. CP leads to fibrosis, which is a common pathological feature and major risk factor for PC [123]. Pancreatic cancer results from dysregulation of oncogenes and tumor suppressor genes, as well as growth factors and their receptors, including epidermal growth factors, vascular endothelial growth factor (VEGF), fibroblast growth factor (FGF), and many cytokines, such as TGF-β, IL-1, IL-6, TNF-α, and IL-8, which modulate pathways involved in growth and differentiation [124, 125]. Shi, *et al.* has showed that VEGF is upregulated by low extracellular PH (acidosis), which occurs frequently around necrotic regions in tumors, and that acidosis activates IL-8 [126]. VEGF and IL-8 are important angiogenic factors in PC [126], and acidosis-promoted upregulation of these genes can be mediated through NF-κB and AP-1 transactivation and cooperation [127].

### Liver

Inflammation in the liver protects this organ from infection and injury, but excessive inflammation may lead to extensive loss of hepatocytes, ischemia-reperfusion injury, metabolic alterations, and eventually permanent hepatic damage [128]. Inflammation can destroy hepatic parenchymal cells, increasing the risk of chronic liver diseases, such as non-alcoholic fatty liver disease (NAFLD) or viral hepatitis. Chronic liver diseases are a leading cause of morbidity and mortality in the US [129].

The liver is the largest solid organ in the body [130], and is a target of both infectious and non-infectious inflammatory pathologies. Infectious inflammation of the liver is mainly caused by microorganisms, such as bacterial products, hepatitis B virus (HBV), or hepatitis C virus (HCV) [131, 132]. Sterile inflammation (SI) is also important in the pathology of many liver diseases, such as alcoholic or nonalcoholic steatohepatitis, drug-induced liver injury, and ischemia/reperfusion [133–135]. In SI, endogenous DAMPs are released to injured tissues and activate immune cells [136]. While pathogen-driven inflammation and SI differ, they share several functional characteristics. Many receptors and pathways can be activated by both PAMPs and DAMPs [137]. TLR4, for example, can be activated by bacterial LPS and cellular HMGB1. Because of the liver's unique vascular supply, PAMPs of intestinal origin and DAMPs from hepatocytes both contribute to inflammation in a variety of diseases. For example, PRR activation by DAMPs and PAMPs can induce production of pro-inflammatory cytokines and immune cell localization to sites of injury. Recognition of DAMPs and PAMPs results in assembly of the inflammasome, a cytosolic protein complex that activates the serine protease caspase-1, followed by activation and secretion of IL-1β and other cytokines. At the same time, Kupffer cell activation and inflammatory cell recruitment leads to production of cytokines and chemokines that promote long-term inflammation, hepatocyte damage, and/or cholestasis [138].

### Lung

Lung inflammatory diseases involve complex interactions among and between structural and immune cells [139]. Lung inflammation results predominantly from tissue exposure to bacterial and viral pathogens, and/or environmental pollutants. Excessive acute inflammation and subsequent lung injury can cause pulmonary fibrosis and impair gas exchange. Unresolved lung injury and chronic inflammation are frequently observed in acute respiratory distress syndrome, cystic fibrosis, chronic obstructive pulmonary disease (COPD), and asthma [140–142]. Approximately 90% of COPD cases are associated with cigarette smoking-induced inflammation in small airways and lung parenchyma [143]. Cigarette smoking is a major risk factor for COPD, which involves both systemic and pulmonary inflammation. Long-term smoking can cause macrophage, neutrophil, and activated T lymphocyte infiltration into airways, and promote production of chemokines, oxygen radicals, proteases, and cytokines, including that of TNF-α, IL-6 and IL-8, in the lung [144].

### Kidney

Kidney inflammation contributes to progressive renal injury, which may lead to glomerulonephritis, end-stage renal disease, or acute or chronic kidney disease (CKD) [145–147]. Approximately 10–12% of the population suffers from CKD, and some 50% of elderly patients show signs of kidney dysfunction, which is associated with high morbidity and mortality [52]. Kidney inflammation is most commonly induced by infection, ischemia/reperfusion, *in situ* immune-complex formation/deposition, or complement pathway dysregulation [145]. CKD and acute kidney injury (AKI) are the most severe types of kidney disease [148]. Interstitial inflammation and tubular injury are commonly observed in acute and chronic kidney injury cases. Renal tubular epithelial cells are likely important promoters of kidney inflammation, secreting a variety of inflammatory cytokines in response to both immune and non-immune factors, and leukocyte infiltration depends on the local presence of these cytokines [146]. Stimuli that can induce kidney injury activate transcription factors (NF-κB or MAPK). These stimuli include cytokines, growth factors, DAMPs, and PAMPs, TLRs, Nod-like receptors (NLRs), and metabolic (high glucose, advanced glycosylation end products) and immune mediators [147].

### Intestinal tract

Acute and chronic inflammatory diseases of the intestine can cause various health issues, and decrease patient quality of life worldwide [149, 150]. The complex, polygenetic inflammatory bowel diseases (IBDs) are characterized by an excessive inflammatory response to gut lumen microbial flora [151]. IBDs mainly include ulcerative colitis (UC) and Crohn disease (CD), but also noninfectious inflammation of the bowel [152, 153]. Idiopathic IBDs, such as CD and UC, are caused by cytokine-driven, non-infectious inflammation of the gut. For example, CD is associated with excessive IFN-γ/IL-17 and IL-12/IL-23 production, while UC is associated with excess IL-13 [153]. Thus, IBD appears to be the result of a dysfunctional interaction between gut bacteria and the mucosal immune system [154]. A key process in the immune system's response to microbes is the recognition of microbial agents via PRRs, including TLRs and nucleotide-binding oligomerization domain containing NLRs, which sense evolutionarily conserved PAMPs [155]. Upon PAMP detection, PRRs activate intracellular signaling pathways that induce production of cytokines and chemokines to promote host resistance to infection. TLR (mainly TLR4) signaling induces NF-κB and MAPK transcription. At the same time, NLRs are also activated through ligand detection [154], in turn activating caspase-1, followed by activation and secretion of IL-1β, interleukin-18 and other cytokines [154].

### Reproductive system

The hallmarks of inflammation are observed during many normal reproductive processes, including menstruation, ovulation, implantation, and parturition [156]. Injury and healing caused by menstruation, ovulation, and parturition trigger the inflammatory cascade. However, initiation and maintenance of inflammatory processes are also important components of many reproductive tract diseases. Damaged tissues locally release inflammatory interleukins, growth factors, cytokines, and prostaglandins, which activate signaling pathways and recruit immune cells (e.g. neutrophils and macrophages) to the site of injury. This process synergistically controls tissue remodeling and repair, but can also induce inflammatory diseases [7]. Inflammatory cytokines, including IL-6, are the primary mediators of inflammation-related reproductive tract diseases, and act via signal transduction pathways such as the MAPK pathway [157, 158].

### Brain

Inflammatory responses occur in the brain in many central nervous system (CNS) diseases, including autoimmune diseases, neurodegenerative diseases like Alzheimer's (AD) and Parkinson's disease (PD), and epilepsy. Inflammatory responses in the brain can enhance neuronal excitability, injure cells, and increase blood-brain barrier permeability to various molecules [159–161]. Inflammation-associated CNS diseases result from activation of the brain's resident immune cells and microglia, which produce pro-inflammatory markers [162]. These inflammation processes also involve both the innate and adaptive immune systems and resemble immune responses to systemic infection. Cytokines and TLRs are major inflammatory mediators in the transition between innate and adaptive. Inflammatory responses in the CNS may also be triggered by endogenous ligands recognized by TLRs. DAMPs, such as heat-shock proteins and extracellular matrix degradation molecules, entering the brain through a damaged blood-brain barrier may initiate inflammatory responses. The CNS inflammatory response is strong in reaction to both infectious agents and brain injury,such as tissue damage observed following ischemic, traumatic, or excitotoxic brain injury, or seizure [160, 163, 164].

## CONCLUSIONS

Inflammation is frequently a key element in the pathological progression of organ disease. Three main pathways, NF-κB, MAPK, and JAK-STAT, play major roles in inflammation, and dysregulation of one or more of these pathways may lead to inflammation-associated disease. A better understanding of inflammatory response pathways and molecular mechanisms will undoubtedly contribute to improved prevention and treatment of inflammatory diseases.

## References

[1] Medzhitov R (2010). Inflammation 2010: new adventures of an old flame. Cell.

[2] Ferrero-Miliani L, Nielsen O, Andersen P, Girardin S (2007). Chronic inflammation: importance of NOD2 and NALP3 in interleukin-1β generation. Clin Exp Immunol.

[3] Nathan C, Ding A (2010). Nonresolving inflammation. Cell.

[4] Zhou Y, Hong Y, Huang H (2016). Triptolide Attenuates Inflammatory Response in Membranous Glomerulo-Nephritis Rat via Downregulation of NF-κB Signaling Pathway. Kidney and Blood Pressure Res.

[5] Takeuchi O, Akira S (2010). Pattern Recognition Receptors and Inflammation. Cell.

[6] Chertov O, Yang D, Howard O, Oppenheim JJ (2000). Leukocyte granule proteins mobilize innate host defenses and adaptive immune responses. Immunol Rev.

[7] Jabbour HN, Sales KJ, Catalano RD, Norman JE (2009). Inflammatory pathways in female reproductive health and disease. Reprod.

[8] Lawrence T (2009). The Nuclear Factor NF-κB Pathway in Inflammation. CSH Perspect Biol.

[9] Libby P (2007). Inflammatory mechanisms: the molecular basis of inflammation and disease. Nutr Rev.

[10] Brusselle G, Bracke K (2014). Targeting immune pathways for therapy in asthma and chronic obstructive pulmonary disease. Annals American Thoracic Society.

[11] Gudkov AV, Komarova EA (2016). p53 and the Carcinogenicity of Chronic Inflammation. CSH Perspect Med.

[12] Seong SY, Matzinger P (2004). Hydrophobicity: an ancient damage-associated molecular pattern that initiates innate immune responses. Nat Rev Immunol.

[13] Ozinsky A, Underhill DM, Fontenot JD, Hajjar AM, Smith KD, Wilson CB, Schroeder L, Aderem A (2000). The repertoire for pattern recognition of pathogens by the innate immune system is defined by cooperation between toll-like receptors. P Natl Acad Sci.

[14] Janeway CA, Medzhitov R (2002). Innate immune recognition. Annu rev of immunol.

[15] Yamamoto M, Takeda K (2010). Current Views of Toll-Like Receptor Signaling Pathways. Gastroenterol Res Pract.

[16] Czerkies M, Kwiatkowska K (2014). Toll-Like Receptors and their Contribution to Innate Immunity: Focus on TLR4 Activation by Lipopolysaccharide. Adv Cell Biol.

[17] Akira S, Takeda K, Kaisho T (2001). Toll-like receptors: critical proteins linking innate and acquired immunity. Nat Immunol.

[18] Adib-Conquy M, Cavaillon JM (2007). Stress molecules in sepsis and systemic inflammatory response syndrome. FEBS let.

[19] Rubartelli A, Lotze MT (2007). Inside, outside, upside down: damage-associated molecular-pattern molecules (DAMPs) and redox. Trends immunol.

[20] Kaminska B (2005). MAPK signalling pathways as molecular targets for anti-inflammatory therapy--from molecular mechanisms to therapeutic benefits. BBA.

[21] Hendrayani SF, Al-Harbi B, Al-Ansari MM, Silva G, Aboussekhra A (2016). The inflammatory/cancer-related IL-6/STAT3/NF-κB positive feedback loop includes AUF1 and maintains the active state of breast myofibroblasts. Oncotarget.

[22] Kyriakis JM, Avruch J (2001). Mammalian mitogen-activated protein kinase signal transduction pathways activated by stress and inflammation. Physiol Rev.

[23] Henríquez-Olguín C, Altamirano F, Valladares D, López JR, Allen PD, Jaimovich E (2015). Altered ROS production, NF-κB activation and interleukin-6 gene expression induced by electrical stimulation in dystrophic mdx skeletal muscle cells. BBA-Mol Basis Disc.

[24] Girard S, Kadhim H, Roy M, Lavoie K, Brochu ME, Larouche A, Sébire G (2009). Role of perinatal inflammation in cerebral palsy. Pediatr neurol.

[25] Moynagh PN (2005). The NF-kB pathway. J Cell Sci.

[26] Hoffmann A, Natoli G, Ghosh G (2006). Transcriptional regulation via the NF-|[kappa]|B signaling module. Oncogene.

[27] Pasparakis M, Luedde T, Schmidt-Supprian M (2006). Dissection of the NF-κB signalling cascade in transgenic and knockout mice. Cell Death Differ.

[28] Basak S, Kim H, Kearns JD, Tergaonkar V, O’Dea E, Werner SL, Benedict CA, Ware CF, Ghosh G, Verma IM (2007). A fourth IκB protein within the NF-κB signaling module. Cell.

[29] Kadhim H, Tabarki B, Verellen G, De Prez C, Rona AM, Sebire G (2001). Inflammatory cytokines in the pathogenesis of periventricular leukomalacia. Neurology.

[30] Hayden MS, Ghosh S (2012). NF-κB, the first quarter-century: remarkable progress and outstanding questions. Gene Dev.

[31] Kaminska B (2005). MAPK signalling pathways as molecular targets for anti-inflammatory therapy--from molecular mechanisms to therapeutic benefits. Biochimica et Biophysica Acta (BBA) - Proteins and Proteomics.

[32] Pearson G, Robinson F, Beers GT, Xu BE, Karandikar M, Berman K, Cobb MH (2001). Mitogen-activated protein (MAP) kinase pathways: regulation and physiological functions. Endocr Rev.

[33] Kim EK, Choi EJ (2010). Pathological roles of MAPK signaling pathways in human diseases. BBA-Mol Basis.

[34] Dhillon AS, Hagan S, Rath O, Kolch W (2007). MAP kinase signalling pathways in cancer. Oncogene.

[35] Sabio G, Davis RJ (2014). TNF, MAP kinase signalling pathways. Semin in Immunol.

[36] Raingeaud J, Whitmarsh AJ, Barrett T, Dérijard B, Davis RJ (1996). MKK3- and MKK6-regulated gene expression is mediated by the p38 mitogen-activated protein kinase signal transduction pathway. Mol Cell Biolog.

[37] O’Shea JJ, Schwartz DM, Villarino AV, Gadina M, Mcinnes IB, Laurence A (2015). The JAK-STAT pathway: impact on human disease and therapeutic intervention. Annu Rev Med.

[38] Walker JG, Smith MD (2005). The Jak-STAT pathway in rheumatoid arthritis. J Rheumatol.

[39] Ivashkiv LB, Hu X (2003). The JAK/STAT pathway in rheumatoid arthritis: pathogenic or protective?. Arthritis Rheumatol.

[40] Boengler K, Hilfiker-Kleiner D, Drexler H, Heusch G, Schulz R (2008). The myocardial JAK/STAT pathway: from protection to failure. Pharmacol Therapeut.

[41] Oeckinghaus A, Hayden MS, Ghosh S (2011). Crosstalk in NF-κB signaling pathways. Nat Immunol.

[42] Iwasaki A, Medzhitov R (2004). Toll-like receptor control of the adaptive immune responses. Nat Immunol.

[43] Opitz B, Van LV Eitel J, Suttorp N (2010). Innate immune recognition in infectious and noninfectious diseases of the lung. American J Resp Crit Care Med.

[44] Rahman I, Adcock IM (2006). Oxidative stress and redox regulation of lung inflammation in COPD. Eur Respir J.

[45] Cesari M, Penninx BW, Newman AB, Kritchevsky SB, Nicklas BJ, Sutton-Tyrrell K, Rubin SM, Ding J, Simonsick EM, Harris TB Inflammatory markers and onset of cardiovascular events. Circulation.

[46] Bhowmik A, Seemungal TA, Sapsford RJ, Wedzicha JA (2000). Relation of sputum inflammatory markers to symptoms and lung function changes in COPD exacerbations. Thorax.

[47] Pecoits-Filho R, Heimbürger O, Bárány P, Suliman M, Fehrman-Ekholm I, Lindholm B, Stenvinkel P (2003). Associations between circulating inflammatory markers and residual renal function in CRF patients. Am J Kidney Dis.

[48] Ross AC (2009). Relationship between Inflammatory Markers, Endothelial Activation Markers, and Carotid Intima-Media Thickness in HIV-Infected Patients Receiving Antiretroviral Therapy. Cli Infect Dis.

[49] Pai JK, Pischon T, Ma J, Manson JE, Hankinson SE, Joshipura K, Curhan GC, Rifai N, Cannuscio CC, Stampfer MJ (2004). Inflammatory markers and the risk of coronary heart disease in men and women. New Engl J Med.

[50] Bautista LE, Vera LM, Arenas IA, Gamarra G (2005). Independent association between inflammatory markers (C-reactive protein, interleukin-6, and TNF-alpha) and essential hypertension. J Hum Hypertens.

[51] Carrero JJ, Yilmaz MI, Lindholm B, Stenvinkel P (2008). Cytokine Dysregulation in Chronic Kidney Disease: How Can We Treat It?. Blood Purificat.

[52] Machowska A, Carrero JJ, Lindholm B, Stenvinkel P (2016). Therapeutics targeting persistent inflammation in chronic kidney disease. Translat Res J La Clin Med.

[53] Goldstein BI, Kemp DE, Soczynska JK, Mcintyre RS (2009). Inflammation and the phenomenology, pathophysiology, comorbidity, and treatment of bipolar disorder: a systematic review of the literature. J Clin Psych.

[54] Miller AH, Maletic V, Raison CL (2009). Inflammation and Its Discontents: The Role of Cytokines in the Pathophysiology of Major Depression. Biol Psych.

[55] Lindahl B, Toss H, Siegbahn A, Venge P, Wallentin L (2000). Markers of myocardial damage and inflammation in relation to long-term mortality in unstable coronary artery disease. FRISC Study Group. Fragmin during Instability in Coronary Artery Disease. New Engl J Med.

[56] Shlipak MG, Fried LF, Crump C, Bleyer AJ, Manolio TA, Tracy RP, Furberg CD, Psaty BM (2003). Elevations of inflammatory and procoagulant biomarkers in elderly persons with renal insufficiency. Circulation.

[57] Gupta J, Mitra N, Kanetsky PA, Devaney J, Wing MR, Reilly M, Shah VO, Balakrishnan VS, Guzman NJ, Girndt M (2012). Association between albuminuria, kidney function, and inflammatory biomarker profile in CKD in CRIC. Clin J Am Soc Nephrolog Cjasn.

[58] Turner MD, Nedjai B, Hurst T, Pennington DJ Cytokines and chemokines: At the crossroads of cell signalling and inflammatory disease. BBA-Mol Cell Res.2014;.

[59] Czaja AJ (2014). Hepatic inflammation and progressive liver fibrosis in chronic liver disease. World J Gastroenter.

[60] Liu Z, Wang Y, Wang Y, Ning Q, Zhang Y, Gong C, Zhao W, Jing G, Wang Q (2016). Dexmedetomidine attenuates inflammatory reaction in the lung tissues of septic mice by activating cholinergic anti-inflammatory pathway. Int Immunopharmacol.

[61] Eckersall PD, Bell R (2010). Acute phase proteins: Biomarkers of infection and inflammation in veterinary medicine. Vet J.

[62] Murata H, Shimada N, Yoshioka M (2004). Current research on acute phase proteins in veterinary diagnosis: an overview. Vet J.

[63] Murakami A, Ohigashi H (2007). Targeting NOX, INOS, COX-2 in inflammatory cells: chemoprevention using food phytochemicals. Int J Cancer.

[64] Lopresti AL, Maker GL, Hood SD, Drummond PD (2014). A review of peripheral biomarkers in major depression: the potential of inflammatory and oxidative stress biomarkers. Prog neuro-psychoph.

[65] Huang W, Tang Y, Li L (2010). HMGB1, a potent proinflammatory cytokine in sepsis. Cytokine.

[66] Schierbeck H, Lundbäck P, Palmblad K, Klevenvall L, Erlandssonharris H, Andersson U, Ottosson L (2011). Monoclonal anti-HMGB1 (high mobility group box chromosomal protein 1) antibody protection in two experimental arthritis models. Mol Med.

[67] Cheng Y, Wang D, Wang B, Li H, Xiong J, Xu S, Chen Q, Tao K, Yang X, Zhu Y (2017). HMGB1 translocation and release mediate cigarette smoke–induced pulmonary inflammation in mice through a TLR4/MyD88-dependent signaling pathway. Mol Biol Cell.

[68] Asavarut P, Zhao H, Gu J, Ma D (2013). The role of HMGB1 in inflammation-mediated organ injury. Acta Anaesthesiol Taiwanica.

[69] Park J, Min JS, Kim B, Chae UB, Yun JW, Choi MS, Kong IK, Chang KT, Lee DS (2015). Mitochondrial ROS govern the LPS-induced pro-inflammatory response in microglia cells by regulating MAPK, NF-κB pathways. Neurosci Lett.

[70] Reuter S, Gupta SC, Chaturvedi MM, Aggarwal BB (2010). Oxidative stress, inflammation, and cancer: how are they linked?. Free Radical Biol Med.

[71] Stramer BM, Mori R, Martin P (2007). The inflammation-fibrosis link? A Jekyll and Hyde role for blood cells during wound repair. J Invest Dermatol.

[72] Van LS Miteva K, Tschöpe C (2014). Crosstalk between fibroblasts and inflammatory cells. Cardiovasc Res.

[73] Robb CT, Regan KH, Dorward DA, Rossi AG (2016). Key mechanisms governing resolution of lung inflammation. Semin Immunopathol.

[74] Nathan C (2006). Neutrophils and immunity: challenges and opportunities. Nat Rev Immunol.

[75] Fujiwara N, Kobayashi K (2005). Macrophages in inflammation. Current Drug Targets Inflam Allergy.

[76] Huang C, Šali A, Stevens RL (1998). Regulation and Function of Mast Cell Proteases in Inflammation. J Clin Immunol.

[77] Aggrey AA, Srivastava K, Ture S, Field DJ, Morrell CN (2013). Platelet induction of the acute-phase response is protective in murine experimental cerebral malaria. J Immunol.

[78] Headland SE, Norling LV (2015). The resolution of inflammation: Principles and challenges. Semin Immunol.

[79] Reville K, Crean JK, Vivers S, Dransfield I, Godson C (2006). Lipoxin A4 redistributes myosin IIA, Cdc42 in macrophages: implications for phagocytosis of apoptotic leukocytes. Jof Immunol.

[80] Serhan CN, Savill J (2005). Resolution of inflammation: the beginning programs the end. Nat Immunol.

[81] Lintermans LL, Stegeman CA, Heeringa P, Abdulahad WH (2014). T cells in vascular inflammatory diseases. Front Immunol.

[82] Sugimoto MA, Sousa LP, Pinho V, Perretti M, Teixeira MM (2016). Resolution of Inflammation: What Controls Its Onset? Front Immuno.

[83] He G, Karin M (2011). NF-κB, STAT3 - key players in liver inflammation and cancer. Cell Res.

[84] Sofi F, Fabbri A, Casini A (2016). Inflammation and Cardiovascular Disease and Protection by the Mediterranean Diet. Mediterranean Diet.

[85] Khan N, Khymenets O, Urpísardà M, Tulipani S, Garciaaloy M, Monagas M, Moracubillos X, Llorach R, Andreslacueva C (2014). Cocoa Polyphenols and Inflammatory Markers of Cardiovascular Disease. Nutrients.

[86] Lloydjones D, Adams RJ, Brown TM, Carnethon M, Dai S, Simone GD, Ferguson TB, Ford E, Furie K, Gillespie C (2010). Heart Disease and Stroke Statistics—2010 Update A Report From the American Heart Association. Circulation.

[87] Mathers CD, Loncar D (2006). Projections of Global Mortality and Burden of Disease from 2002 to 2030. Plos Med.

[88] Packard RRS, Peter L (2008). Inflammation in atherosclerosis: from vascular biology to biomarker discovery and risk prediction. Clin Chem.

[89] Libby P (2012). History of Discovery: Inflammation in Atherosclerosis. Arteriosclerosis Thrombosis Vascular Biology.

[90] Libby P (2002). Atherosclerosis in Inflammation. Nature.

[91] Libby P, Okamoto Y, Rocha VZ, Folco E (2010). Inflammation in atherosclerosis: transition from theory to practice. Circulation J.

[92] Glezeva N, Baugh JA (2014). Role of inflammation in the pathogenesis of heart failure with preserved ejection fraction and its potential as a therapeutic target. Heart Fail Rev.

[93] Askevold ET, Gullestad L, Dahl CP, Yndestad A, Ueland T, Aukrust P (2014). Interleukin-6 signaling, soluble glycoprotein 130, and inflammation in heart failure. Curr Heart Fail Reports.

[94] Jennings RB, Murry CE, Steenbergen C, Reimer KA (1990). Development of cell injury in sustained acute ischemia. Circulation.

[95] Pfeffer MA, Braunwald E (1990). Ventricular remodeling after myocardial infarction. Experimental observations and clinical implications. Circulation.

[96] Opie LH, Commerford PJ, Gersh BJ, Pfeffer MA (2006). Controversies in ventricular remodelling. Lancet.

[97] Frangogiannis NG (2008). The immune system and cardiac repair. Pharmacol Res.

[98] Beg AA (2002). Endogenous ligands of Toll-like receptors: implications for regulating inflammatory and immune responses. Trends Immunol.

[99] Frantz S, Ertl G, Bauersachs J (2007). Mechanisms of Disease: Toll-like receptors in cardiovascular disease. Nat Clin Pract Card.

[100] Nijmeijer R, Lagrand WK, Visser CA, Meijer CJ, Niessen HW, Hack CE (2001). CRP, a major culprit in complement-mediated tissue damage in acute myocardial infarction?. Int Immunopharmacol.

[101] Giordano FJ (2005). Oxygen, oxidative stress, hypoxia, and heart failure. J Clin Invest.

[102] Griendling KK, Fitzgerald GA (2003). Oxidative Stress and Cardiovascular Injury: Part I: Basic Mechanisms and In Vivo Monitoring of ROS. Circulation.

[103] Hall G, Hasday JD, Rogers TB (2006). Regulating the regulator: NF-κB signaling in heart. J Mol Cell Cardiol.

[104] Gerard C, Rollins BJ (2001). Chemokines and disease. Nat Immunol.

[105] Moser B, Loetscher P (2001). Lymphocyte traffic control by chemokines. Nat Immunol.

[106] Kaur K, Dhingra S, Slezak J, Sharma AK, Bajaj A, Singal PK (2009). Biology of TNFα and IL-10, and their imbalance in heart failure. Heart Fail Rev.

[107] Frangogiannis NG (2014). Targeting the transforming growth factor (TGF)-β cascade in the remodeling heart: Benefits and perils. J Mol Cell Cardiol.

[108] Booth GL, Kapral MK, Fung K, Tu JV (2006). Relation between age and cardiovascular disease in men and women with diabetes compared with non-diabetic people: a population-based retrospective cohort study. Lancet.

[109] Low Wang CC, Hess CN, Hiatt WR, Goldfine AB (2016). Clinical Update: Cardiovascular Disease in Diabetes Mellitus: Atherosclerotic Cardiovascular Disease and Heart Failure in Type 2 Diabetes Mellitus - Mechanisms, Management, and Clinical Considerations. Circulation.

[110] Turner MD (2017). The identification of TNFR5 as a therapeutic target in diabetes. Taylor Francis.

[111] Pradeep T, Haranath C (2014). A Review on Diabetes Mellitus Type II. Int J Pharma Res Rev.

[112] Larsen CM, Faulenbach M, Vaag A, Vølund A, Ehses JA, Seifert B, Mandrup-Poulsen T, Donath MY (2007). Interleukin-1–receptor antagonist in type 2 diabetes mellitus. New Engl J Med.

[113] Donath MY, Schumann DM, Faulenbach M, Ellingsgaard H, Perren A, Ehses JA (2008). Islet inflammation in type 2 diabetes. Diabetes care.

[114] Esser N, Legrand-Poels S, Piette J, Scheen AJ, Paquot N (2014). Inflammation as a link between obesity, metabolic syndrome and type 2 diabetes. Diabetes Res Clin Pr.

[115] Dinarello CA, Donath MY, Mandrup-Poulsen T (2010). Role of IL-1β in type 2 diabetes. Curr Opin Endocrinol.

[116] Donath MY, Shoelson SE (2011). Type 2 diabetes as an inflammatory disease. Nat Rev Immunol.

[117] Manohar M, Verma AK, Venkateshaiah SU, Sanders NL, Mishra A (2017). Pathogenic mechanisms of pancreatitis. World J Gastr Pharmacol Therapeut.

[118] Yadav D, Lowenfels AB (2013). The epidemiology of pancreatitis and pancreatic cancer. Gastroenterology.

[119] Zheng L, Xue J, Jaffee EM, Habtezion A (2013). Role of immune cells and immune-based therapies in pancreatitis and pancreatic ductal adenocarcinoma. Gastroenterology.

[120] Zhang H, Neuhöfer P, Song L, Rabe B, Lesina M, Kurkowski MU, Treiber M, Wartmann T, Regnér S, Thorlacius H (2013). IL-6 trans-signaling promotes pancreatitis-associated lung injury and lethality. J Clin Invest.

[121] Waddell N, Pajic M, Patch AM, Chang DK, Kassahn KS, Bailey P, Johns AL, Miller D, Nones K, Quek K (2015). Whole genomes redefine the mutational landscape of pancreatic cancer. Nature.

[122] Ling S, Feng T, Jia K, Tian YU, Yan LI (2014). Inflammation to cancer: The molecular biology in the pancreas (Review). Oncol Lett.

[123] Inflammation Whitcomb DC., Cancer V (2004). Chronic pancreatitis and pancreatic cancer. Am J Physiol-Gastr L.

[124] Li D, Xie K, Wolff R, Abbruzzese JL (2004). Pancreatic cancer. Lancet.

[125] Goggins M, Hruban RH, Kern SE (2000). BRCA2 is inactivated late in the development of pancreatic intraepithelial neoplasia: evidence and implications. Am J pathoL.

[126] Shi Q, Le X Wang B, Abbruzzese JL, Xiong Q, He Y, Xie K (2001). Regulation of vascular endothelial growth factor expression by acidosis in human cancer cells. Oncogene.

[127] Xie K (2001). Interleukin-8 and human cancer biology. Cytokine Growth F R.

[128] Brenner C, Galluzzi L, Kepp O, Kroemer G (2013). Decoding cell death signals in liver inflammation. J Hepatol.

[129] Leitão HS, Doblas S, Garteiser P, D’Assignies G, Paradis V, Mouri F, Geraldes CF, Ronot M, Van Beers BE (2016). Hepatic Fibrosis, Inflammation, and Steatosis: Influence on the MR Viscoelastic and Diffusion Parameters in Patients with Chronic Liver Disease. Radiology.

[130] Ramadori G, Moriconi F, Malik I, Dudas J (2008). Physiology and pathophysiology of liver inflammation, damage and repair. J Physiol Pharmacol.

[131] Pawlotsky JM (2004). Pathophysiology of hepatitis C virus infection and related liver disease. Trends in Microbiol.

[132] Dunn C, Brunetto M, Reynolds G, Christophides T, Kennedy PT, Lampertico P, Das A, Lopes AR, Borrow P, Williams K, Humphreys E, Afford S, Adams DH (2007). Cytokines induced during chronic hepatitis B virus infection promote a pathway for NK cell–mediated liver damage. J Exp Med.

[133] Gao B, Seki E, Brenner DA, Friedman S, Cohen JI, Nagy L, Szabo G, Zakhari S (2011). Innate immunity in alcoholic liver disease. Am J Physiol Gastrointest Liver Physiol.

[134] Brenner DA, Seki E, Taura K, Kisseleva T, Deminicis S, Iwaisako K, Inokuchi S, Schnabl B, Oesterreicher CH, Yong HP (2011). Non-alcoholic steatohepatitis-induced fibrosis: Toll-like receptors, reactive oxygen species and Jun N-terminal kinase. Hepatol Res.

[135] Maher JJ (2009). DAMPs ramp up drug toxicity. J Clin Invest.

[136] Kubes P, Mehal WZ (2012). Sterile Inflammation in the Liver. Gastroenterology.

[137] Poltorak A, He X, Smirnova I, Liu MY, Huffel CV, Du X, Birdwell D, Alejos E, Silva M, Galanos C (1998). Defective LPS Signaling in C3H/HeJ and C57BL/10ScCr Mice: Mutations in Tlr4 Gene. science.

[138] Szabo G, Mandrekar P, Dolganiuc A (2007). Innate immune response and hepatic inflammation. Seminars in Liver Disease.

[139] Walford HH, Doherty TA (2013). STAT6 and lung inflammation. Jakstat.

[140] Leitch AE, Duffin R, Haslett C, Rossi AG (2008). Relevance of granulocyte apoptosis to resolution of inflammation at the respiratory mucosa. Mucosal Immunol.

[141] Brusselle G, Bracke K (2014). Targeting immune pathways for therapy in asthma and chronic obstructive pulmonary disease. Annals of the American Thoracic Society.

[142] Wong J, Magun BE, Wood LJ (2016). Lung inflammation caused by inhaled toxicants: a review. Int J of Copd.

[143] Mroz RM, Noparlik J, Chyczewska E, Braszko JJ, Holownia A (2007). Molecular basis of chronic inflammation in lung diseases: new therapeutic approach. J Physiol Pharmacol.

[144] Kawayama T, Kinoshita T, Matsunaga K, Kobayashi A, Hayamizu T, Johnson M, Hoshino T (2016). Responsiveness of blood and sputum inflammatory cells in Japanese COPD patients, non-COPD smoking controls, and non-COPD nonsmoking controls. Int J Copd.

[145] Ernandez T, Mayadas TN (2016). The Changing Landscape of Renal Inflammation. Trends Mol Med.

[146] Poveda J, Sanz AB, Rayegomateos S, Ruizortega M, Carrasco S, Ortiz A, Sanchezniño MD (2016). NFκBiz protein downregulation in acute kidney injury: Modulation of inflammation and survival in tubular cells. BBA - Mol Basis Dis.

[147] Sanz A, Sanchez-Niño M, Ramos A, Moreno J, Santamaria B, Ruiz-Ortega M, Egido J, Ortiz A (2010). NF-kappaB in renal inflammation. J Am Soc Nephrol.

[148] Chawla LS, Kimmel PL (2012). Acute kidney injury and chronic kidney disease: an integrated clinical syndrome. Kidney Int.

[149] Sanchez MIP, Bercik P (2011). Epidemiology and burden of chronic constipation. Can J Gastroenterol.

[150] Hunt R, Quigley E, Abbas Z, Eliakim A, Emmanuel A, Goh KL, Guarner F, Katelaris P, Smout A, Umar M (2014). Coping with common gastrointestinal symptoms in the community: a global perspective on heartburn, constipation, bloating, and abdominal pain/discomfort May 2013. J Clin Gastroenterol.

[151] Mcguckin MA, Eri R, Simms LA, Florin TH, Radford-Smith G (2009). Intestinal barrier dysfunction in inflammatory bowel diseases. Inflamm Bowel Dis.

[152] Cario E, Podolsky DK (2000). Differential Alteration in Intestinal Epithelial Cell Expression of Toll-Like Receptor 3 (TLR3) and TLR4 in Inflammatory Bowel Disease. Infection Immunity.

[153] Strober W, Fuss I, Mannon P (2007). The fundamental basis of inflammatory bowel disease. J Clin Invest.

[154] Fukata M, Arditi M (2013). The role of pattern recognition receptors in intestinal inflammation. Mucosal Immunol.

[155] Thompson MR, Kaminski JJ, Kurt-Jones EA, Fitzgerald KA (2011). Pattern Recognition Receptors and the Innate Immune Response to Viral Infection. Viruses.

[156] Goswami B, Rajappa M, Sharma M, Sharma A (2008). Inflammation: its role and interplay in the development of cancer, with special focus on gynecological malignancies. Int J Gynecol Cancer.

[157] Levi M, van der Poll T (2005). Two-way interactions between inflammation and coagulation. Trends Cardiovascular Med.

[158] Sampson MT, Kakkar AK (2002). Coagulation proteases and human cancer. Biochem Soc T.

[159] Nelson PT, Soma LA, Lavi E (2002). Microglia in diseases of the central nervous system. Annals Med.

[160] Vezzani A, Granata T (2005). Brain Inflammation in Epilepsy: Experimental and Clinical Evidence. Epilepsia.

[161] Block ML, Zecca L, Hong JS (2007). Microglia-mediated neurotoxicity: uncovering the molecular mechanisms. Nat Rev Neuro.

[162] Ekdahl CT, Claasen JH, Bonde S, Kokaia Z, Lindvall O (2003). Inflammation is detrimental for neurogenesis in adult brain. P Natl Acad of Sci USA.

[163] Koltsakis GC, Stamatelos AM (2001). Cytokines and acute neurodegeneration. Nat Rev Neuro.

[164] Jankowsky JL, Patterson PH (2001). The role of cytokines and growth factors in seizures and their sequelae. Prog Neurobio.

